# Direct and Sensitive Electrochemical Detection of Bisphenol A in Complex Environmental Samples Using a Simple and Convenient Nanochannel-Modified Electrode

**DOI:** 10.3389/fchem.2022.900282

**Published:** 2022-05-26

**Authors:** Jie Huang, Tongtong Zhang, Guotao Dong, Shanshan Zhu, Fei Yan, Jiyang Liu

**Affiliations:** ^1^ Key Laboratory of Surface and Interface Science of Polymer Materials of Zhejiang Province, Department of Chemistry, Zhejiang Sci-Tech University, Hangzhou, China; ^2^ Heihe Water Resources and Ecological Protection Research Center, Lanzhou, China; ^3^ Department of Hepatobiliary and Pancreatic Surgery, The Center for Integrated Oncology and Precision Medicine, Affiliated Hangzhou First People’s Hospital, Zhejiang University School of Medicine, Hangzhou, China

**Keywords:** electrochemical sensing, bisphenol, nanochannel, direct and sensitive detection, complex environmental samples

## Abstract

Rapid, convenient, and sensitive detection of Bisphenol A (BPA) in complex environmental samples without the need for tedious pre-treatment is crucial for assessing potential health risks. Herein, we present an electrochemical sensing platform using a simple nanochannel-modified electrode, which enables the direct and sensitive detection of BPA in complex samples. A vertically ordered mesoporous silica-nanochannel film (VMSF) with high-density nanochannels is rapidly and stably grown on the surface of a electrochemically activated glassy carbon electrode (p-GCE) by using the electrochemically assisted self-assembly (EASA) method. The high antifouling capability of the VMSF/p-GCE sensor is proven by investigating the electrochemical behavior of BPA in the presence of model coexisting interfering molecules including amylum, protein, surfactant, and humic acid. The VMSF/p-GCE sensor can sensitively detect BPA ranged from 50 to 1.0 μM and 1.0–10.0 μM, with low detection limits (15 nM). Owing to the electrocatalytic performance and high potential resolution of p-GCE, the sensor exhibits high selectivity for BPA detection in the presence of common environmental pollutants, including bisphenol S (BPS), catechol (CC), hydroquinone (HQ), and 4-nitrophenol (4-NP). In combination with the good antifouling property of the VMSF, direct detection of BPA in environmental water samples and soil leaching solution (SLS) is also realized without separation pretreatment. The developed VMSF/p-GCE sensor demonstrated advantages of simple structure, high sensitivity, good antifouling performance, and great potential in direct electroanalysis of endocrine-disrupting compounds in complex samples.

## Introduction

Bisphenol A [2,2′-bis(4-hydroxyphenyl) propane, BPA] is one of the most widely used chemical raw materials in the world, which is commonly used as a monomer for the production of polymers (e.g. polycarbonate, polyphenylene ether resins, unsaturated polyester resins, etc.) or as an ingredient for the production of fine chemicals (e.g. plasticizers, flame retardants, antioxidants, heat stabilizers, rubber antioxidants, pesticides, coatings, etc.). (Zhang et al., 2021) However, BPA is structurally similar to endocrine hormones such as estradiol and diethylstilbestrol, which helps it bind to estrogen receptors as a kind of endocrine-disrupting compound (EDC). (Takayanagi et al., 2006) It has been proven that overexposure to BPA can harm the endocrine system, nervous system, and immune system in humans and animals and significantly increase the incidence of many cancers (e.g. ovarian cancer, prostate cancer, and leukemia). (Li et al., 2017; Zhang et al., 2018; Bilal et al., 2019; Md Younus et al., 2020) Although many countries have enacted specific laws and regulations on the prohibition of BPA in baby bottles and other food-related containers (Authority, 2006), BPA molecules still widely enter the environment through dust or sewage during production and transportation owing to the widespread use of BPA-related products. (Wetherill et al., 2007; Md Younus et al., 2020) Therefore, rapid, convenient, and sensitive detection of BPA in environmental samples is important for assessing BPA exposure and potential health risks.

Until now, methods for quantitative analysis of BPA include gas chromatography–mass spectrometry (GC-MS) (Correia-Sá et al., 2018; Wang et al., 2021a), high-performance liquid chromatography (HPLC) (Lee et al., 2017), fluorescence spectroscopy (FL) (Wang et al., 2020a), surface-enhanced Raman spectroscopy (SERS) (Chung et al., 2015; Yang et al., 2018; Li et al., 2021), and colorimetry (Lee et al., 2019). However, the detection strategies often suffer from expensive instruments, tedious pretreatment, and high operational requirements. (Cui et al., 2021; Deng et al., 2021; Duan et al., 2021; Wan et al., 2021) On the other hand, the current methods are often only able to detect simple samples with low matrix effects, such as spring water and baby bottle extracts (Kumar Naik et al., 2022; Lei et al., 2022; Rajendran et al., 2022). There are still great challenges in the direct analysis of BPA in complex samples (e.g. environmental water, soil leaching solution, and biological samples). Fast and convenient BPA analysis methods that can realize direct analysis of complex samples are urgently needed.

Electrochemical sensors could offer a unique combination of key merits including simple instrument, convenient operation, and high sensitivity. (Liu et al., 2020; Sabbaghan et al., 2021; Xuan et al., 2021; Ghalkhani and Sohouli, 2022) The electroactive phenolic hydroxyl groups in BPA enable its detection by electrochemical sensing. To improve the detection sensitivity, researchers have used a variety of materials to modify the working electrode, including metal or metal oxide nanoparticles (Ashraf et al., 2020; Wang et al., 2020b; Yang et al., 2022), carbon materials (Yasri et al., 2015; Alam and Deen, 2020; Zhu et al., 2020; Ponnada et al., 2022; Wang et al., 2022), ionic liquids (Wang et al., 2018; Wang et al., 2021b), molecularly imprinted polymers (Beduk et al., 2020; Zhang et al., 2021), metals and covalent organic frameworks (Zhang et al., 2018; Pang et al., 2020), and aptamers (Hadi et al., 2016; Jun et al., 2020). However, these modified electrodes often require expensive reagents/materials or complicated synthesis processes. On the other hand, severe matrix effects in complex samples can passivate the electrode, leading to reduced stability and accuracy. Therefore, an electrochemical sensor with simple electrode structure and antifouling performance is highly desirable to realize the direct analysis of BPA in complex environmental samples without the need for tedious pretreatment.

In recent years, the vertically ordered mesoporous silica-nanochannel film (VMSF) has attracted extensive attention owing to its unique structure and characteristics. The VMSF has highly ordered and uniform nanochannels (usually 2–3 nm in diameter), high porosity (∼75,000 pore/μm (Takayanagi et al., 2006)), and ultrathin nanoscale thickness (commonly 50–200 nm). (Herzog et al., 2013; Nasir et al., 2016; Nasir et al., 2018; Li et al., 2019a; Ding et al., 2020; Ma et al., 2020; Yan et al., 2020; Yan et al., 2021a; Yan et al., 2021b; Wang et al., 2021c; Xiao et al., 2021; Xuan et al., 2021; Zhou et al., 2022) On the one hand, high-density nanochannels offer good permeability. On the other hand, the ultrasmall nanopore structure of the VMSF exhibits remarkable size and charge selectivity. Thus, the VMSF can effectively exclude large-sized substances (e.g. particle, microorganism or cell) or macromolecules (e.g. protein, polysaccharides, and DNA) in complex matrices, leading to high antifouling ability. In addition, the enrichment of small molecules with positive charges by negatively charged nanochannels also significantly improves the detection sensitivity. Thus, the VMSF-modified electrodes show great potential in direct and sensitive detection of redox small molecules in complex samples.

In this work, we demonstrate an electrochemical platform based on the integration of the VMSF on the surface of electrochemically activated glassy carbon electrode (p-GCE), which enables the direct and rapid detection of BPA in complex environmental samples. The abundant active edge sites (defects, oxygen-containing functional groups, etc.) of p-GCE endow it with good electrochemical and electrocatalytic activities. The VMSF was rapidly and stably grown on p-GCE by using the electrochemically assisted self-assembly (EASA) method. High antifouling capability of the VMSF/p-GCE sensor is proven by investigating the electrochemical behavior of BPA in the presence of model coexisting interfering molecules such as proteins, surfactants, and humic acids. As the proof-of-concept demonstrations, direct, rapid, and sensitive detection of BPA in environmental water samples and soil leaching solutions is realized without the usual need of tedious pretreatment.

## Materials and Methods

### Chemicals and Materials

Tetraethoxysilane (TEOS), cetyltrimethylammonium bromide (CTAB), potassium ferricyanide (K_3_ [Fe(CN)_6_]), potassium ferrocyanide (K_4_ [Fe(CN)_6_]), sodium phosphate monobasic dihydrate (NaH_2_PO_4_.2H_2_O), sodium dodecyl sulfate (SDS), amylum, humic acid (HA), sodium phosphate dibasic dodecahydrate (Na_2_HPO_4_.12H_2_O), and catechol (CC) were purchased from Aladdin. Bisphenol A (BPA), hydroquinone (HQ), bisphenol S (BPS), and 4-nitrophenol (4-NP) were purchased from Macklin. Hexaammineruthenium (III) chloride (Ru(NH_3_)_6_Cl_3_) and bovine serum albumin (BSA) were purchased from Sigma Aldrich. Ethanol was obtained from Hangzhou Shuanglin Chemical reagent. Calcium chloride (CaCl_2_), potassium chloride (KCl), sodium chloride (NaCl), magnesium sulfate (MgSO_4_), and sodium nitrate (NaNO_3_) were purchased from the Hangzhou Gaojing Fine Chemical Industry. Environmental water samples were obtained from the lake of Zhejiang Sci-Tech University (Hangzhou, China). Soil leaching solution (SLS) was obtained by leaching the soil (1 g from lawn of Zhejiang Sci-Tech University) in 100 ml ultrapure water. All chemicals and reagents were of analytical grade and used as received without further purification. Ultrapure water (18.2 MΩ cm) was used to prepare all aqueous solutions throughout this work.

### Measurements and Instrumentation

Transmission electron microscopy (TEM) images were obtained at an acceleration voltage of 100 kV on a HT7700 transmission electron microscope (Hitachi, Japan). Before TEM measurement, the VMSF was gently scraped from the p-GCE surface and dispersed in ethanol by ultrasonication. Then, VMSF dispersion was dropped onto the copper grids. All electrochemical experiments including cyclic voltammetry (CV), electrochemical impedance spectroscopy (EIS), and differential pulse voltammetry (DPV) were conducted on an Autolab PGSTAT302N electrochemical workstation (Metrohm, Switzerland). A typical three-electrode system was adopted including bare or modified GCE as the working electrode, an Ag/AgCl electrode (saturated KCl) as the reference electrode, and a platinum wire electrode as the counter electrode. The scan rate in CV is 50 mV/s, unless particularly indicated. For DPV measurements, the step, modulation amplitude, modulation time, and interval time were 0.005 V, 0.025 V, 0.05 s, and 0.2 s, respectively.

### Preparation of p-GCE

The GCE (d = 3 mm) was first polished with 0.3 and 0.05 μm alumina power. The electrode was then sequentially cleaned by sonication in ethanol and ultrapure water and dried under nitrogen flow. Electrochemical activation includes anodic oxidation at high voltage and cathodic reduction at low voltage. Briefly, a constant potential (+1.8 V) was applied on the GCE for 300 s followed with a cyclic voltammetry scan (−1.3–1.25 V, scan segments: 6) in phosphate-buffered solution (PBS, 0.1 M, pH 5). Then, the obtained p-GCE was washed with ultrapure water and dried under nitrogen flow.

### Preparation of the VMSF on p-GCE

The VMSF was grown on p-GCE by using electrochemically assisted self-assembly (EASA) methods as previously reported. (Walcarius et al., 2007) Typically, NaNO_3_ (20 ml, 0.1 M, pH = 2.6) and ethanol (20 ml) were first mixed (v:v = 1:1). Then, CTAB (1.585 g) and TEOS (2.833 g) were subsequently added under stirring. The aforementioned mixture was further stirred for 2.5 h to prehydrolyze TEOS to obtain the precursor solution. After p-GCE was immersed in the precursor solution, growth of the VMSF was performed by applying a cathodic current (−52.2 μA) to p-GCE for 10 s. Then, the obtained electrode was quickly removed from the precursor solution, followed by thorough rinsing with ultrapure water and dried under a N_2_ stream. After further aging at 80°C overnight, the modified electrode containing surfactant micelles (SMs) inside the nanochannels was obtained and termed as SM@VMSF/p-GCE. The removal of SMs could be realized by treating SM@VMSF/p-GCE with a 0.1 M HCl-ethanol solution under stirring for 5 min. The resulting electrode with open nanochannels was termed as VMSF/p-GCE.

### Electrochemical Detection of BPA

Phosphate buffer solution (PBS) (0.1 M, pH = 6) was applied as the buffer for the detection of BPA. The electrochemical responses of different concentration of BPA were recorded using CV or DPV. For real sample analysis, environmental water samples were diluted using the buffer by a factor of 10 without other pretreatments, and the soil was dispersed in the buffer to form a suspension of 1 mg/ml, whose supernatant was further adopted. Then, BPA detection in complex real samples was achieved by the VMSF/p-GCE platform.

## Results and Discussion

### Facile Equipment of the VMSF on p-GCE and Characterization

**FIGURE 1:**
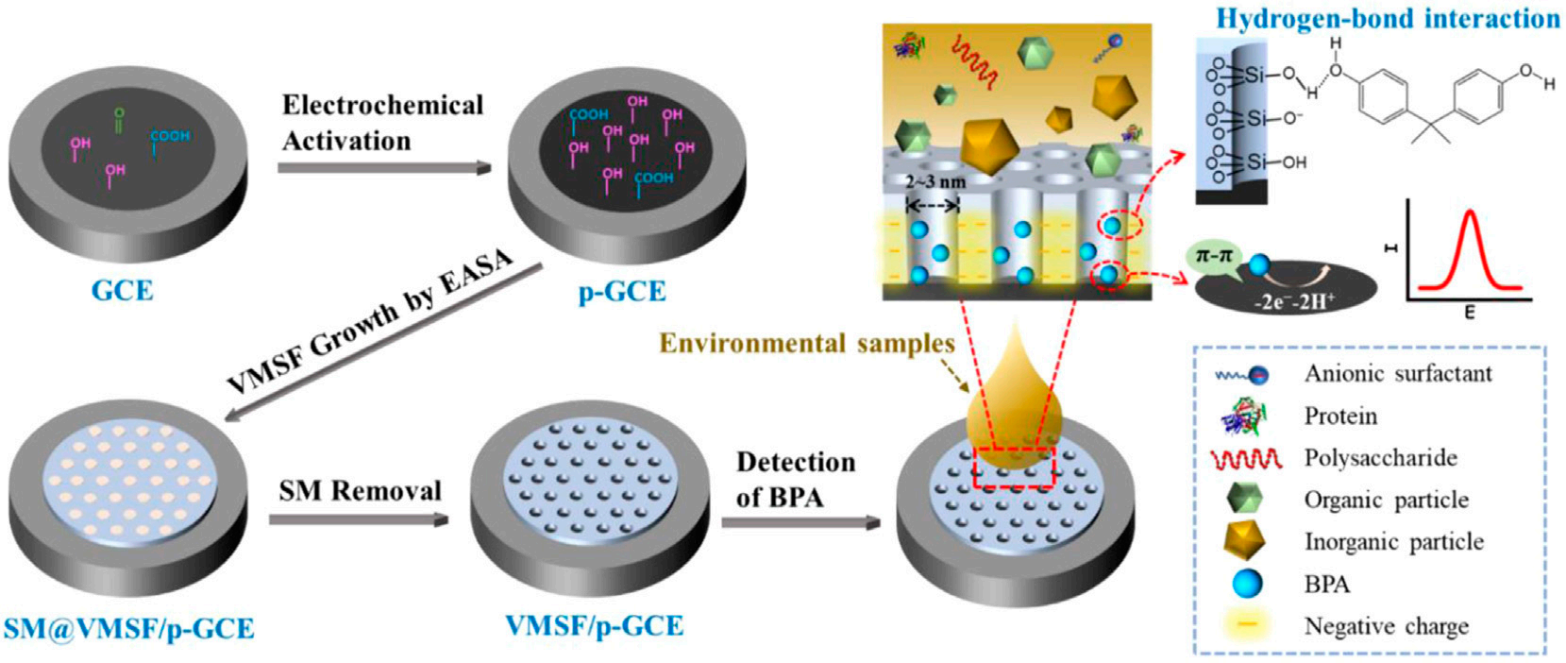
Schematic illustration for the preparation of VMSF/p-GCE and the direct detection of BPA in complex environmental samples.

As illustrated in Figure 1, the electrochemically activated GCE (p-GCE) is applied as the supporting electrode to grow the vertically ordered mesoporous silica-nanochannel film (VMSF). Compared with the GCE, p-GCE has been proven to possess abundant active sites including edge plane sites, defects, and oxygen-containing functional groups. (Li et al., 2019b) These sites can not only enhance the adsorption of electroactive organic molecules (e.g. through electrostatic interaction, hydrogen bonding, etc) but also promote the electron transfer reaction, demonstrating attractive electrocatalytic activity. (Shi and Shiu, 2002) In addition, oxygenated groups on the surface of the p-GCE electrode (e.g. hydroxyl groups) can react with the silanol group so that the VMSF can stably bind on the electrode surface. The VMSF was rapidly and stably grown on p-GCE by the electrochemically assisted self-assembly (EASA) method. The EASA method is a convenient strategy for the fast preparation of the VMSF within 10 s. The principle is to apply a negative voltage on the electrode to promote the reduction of water to generate hydroxyl ions. Then, the pH gradient generated on the surface of the electrode promotes the self-assembly and condensation reactions of siloxanes around surfactant micelles (SMs). After VMSF growth, SMs block nanochannels (SM@VMSF/p-GCE). After further removal of SMs, an electrode with open nanochannels can be obtained (VMSF/p-GCE).

The CV curves of GCE, p-GCE, and VMSF/p-GCE recorded in pure PBS (0.1 M, pH = 6) without any electroactive species are shown in Supplementary Figure S1A (in supporting information-SI). Supplementary Figure S1B (SI) gives the relationship between the peak current obtained on GCE *vs*. the square root of scan rate. The non-Faradaic double layer capacitance (*C*_dl_) can act as quantitative indicator of the surface area that is accessible to the electrolyte ions, which can indicate the electrochemical active surface area (ECSA) of electrodes. (Wei et al., 2019) A notably increased capacitive current is observed on p-GCE compared with that of the GCE (about ∼4 fold increasing), suggesting an enlarged ECSA owing to the generation of a thick porous layer during the electrochemical activation process. (Shi and Shiu, 2002) Interestingly, the ECSA of VMSF/p-GCE only decreases very slightly after the covering of the VMSF. On the other hand, a pair of redox peaks near ∼0 V is observed on p-GCE and VMSF/p-GCE, which are resulted from the conversion between surface-bonded quinone and hydroquinone generated during the electrochemical polarization pretreatment. (Engstrom and Strasser, 1984) Furthermore, p-GCE and VMSF/p-GCE demonstrate an improved electroanalytical reactivity proven by the larger decomposition currents and reduced decomposition potentials for both the anodic and cathodic limits. The exact ECSA of the GCE can be calculated using a reversible probe K_3_ [Fe(CN)_6_] by Randles–Sevcik equation. (Alam and Deen, 2020) The ECSA of the GCE is calculated to be 0.0584 cm^2^. For comparison, the ECSA of p-GCE and VMSF/p-GCE are 0.222 and 0.204 cm^2^, respectively. Thus, the electrochemical polarization increases the active area, and the equipment of the VMSF on p-GCE does not significantly decrease the active area of the electrode.

**FIGURE 2:**
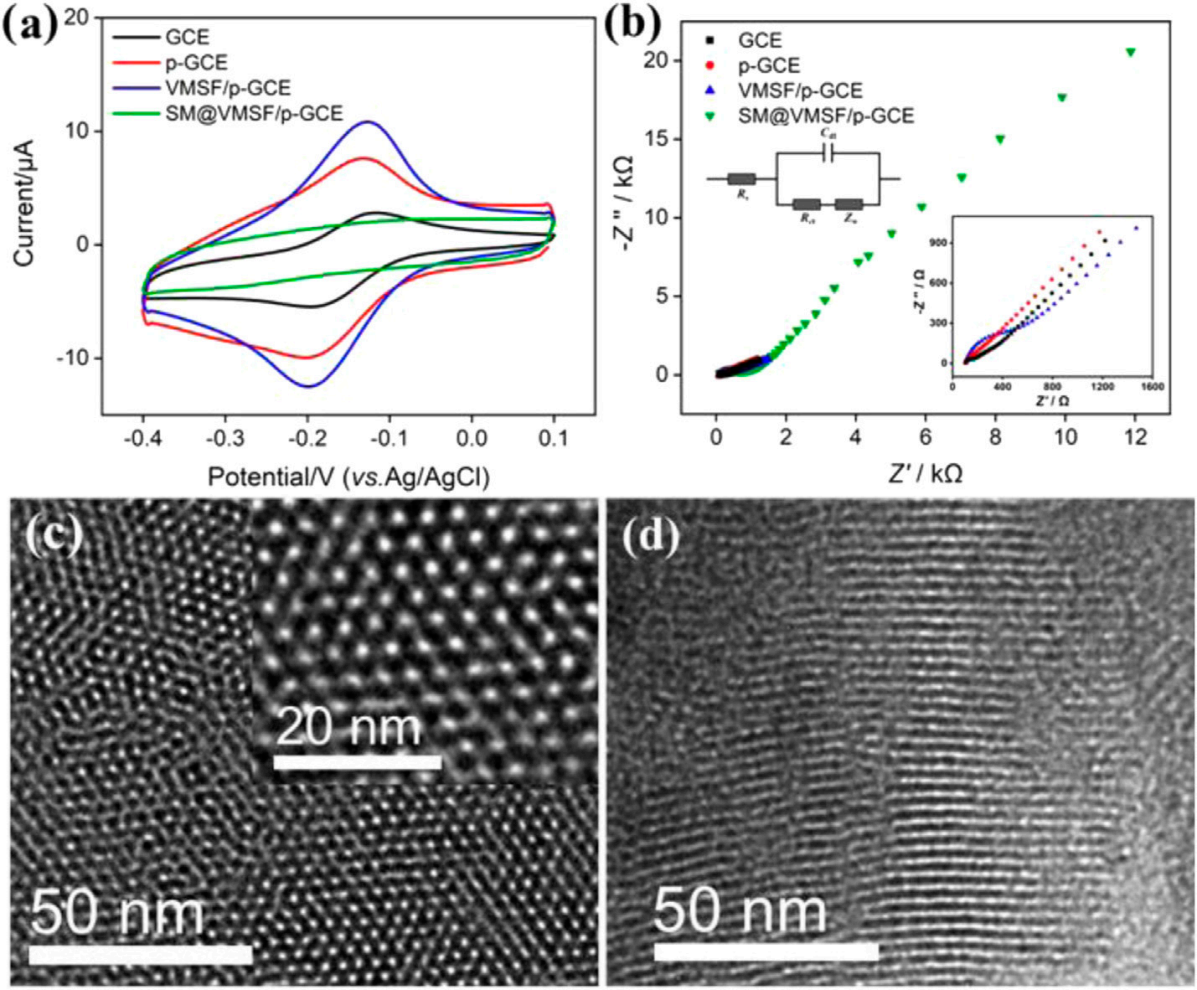
**(A)** CV curves obtained on GCE, p-GCE, VMSF/p-GCE, and SM@VMSF/p-GCE in PBS (0.01 M, pH = 7.4) containing Ru(NH_3_)_6_^3+^ (0.5 mM). **(B)** EIS plots obtained in KCl (0.1 M) containing Fe(CN)_6_^3/4−^ (2.5 mM). Left and right insets are the equivalent circuit and magnified EIS plots, respectively. **(C–D)** Top-view, **(C)** and cross-sectional view **(D)** of TEM images of the VMSF. The inset is the magnified image.

The integrity and permeability of the VMSF were investigated by electrochemical methods. The electrochemical signals of the standard redox probe (Ru(NH_3_)_6_^3+^) on different electrodes including the GCE, p-GCE, VMSF/p-GCE, and SM@VMSF/p-GCE are shown in Figure 2A. The anodic peak potential (*E*_pa_), cathodic peak potential (*E*_pc_), peak-to-peak separation (Δ*E*), anodic peak current (*I*_pa_), and cathodic peak current (*I*_pc_) of all the electrodes are demonstrated in Supplementary Table S1 (SI). As shown, GCE shows a pair of redox peaks with an Δ*E*_p_ of 65.92 mV. For p-GCE, an increase in the peak current (*I*_pa_, 6.813 μA; *I*_pc_, −6.022 μA) and a decrease in the Δ*E*_p_ (63.58 mV) are observed, indicating a faster electron transfer and enlarged ECSA. In the presence of hydrophobic CTAB micelles in the VMSF, there is no Faraday current response at SM@VMSF/p-GCE, indicating that the mass transfer of the hydrophilic probe to the electrode surface is inhibited. The failed electron transfer between the probe and the electrode proves that the VMSF grown on p-GCE is intact with no defects. When the SM is removed and p-GCE is modified with open nanochannels, an increased Δ*E*_p_ of VMSF/p-GCE (65.92 mV) is observed compared with that of p-GCE. However, VMSF/p-GCE demonstrates an enhanced peak current (*I*_pa_, 10.18 μA; *I*_pc_, −9.449 μA). This can be attributed to the strong electrostatic interaction between negatively charged silanols (p*K*_a_ of ∼2) on the nanochannel surface and positively charged Ru(NH_3_)_6_^3+^ at high pH and low ionic strength conditions. (Yan et al., 2020).

Consistent results were obtained from electrochemical impedance spectroscopy (EIS) in the presence of a standard anionic probe (Fe(CN)_6_^3/4−^). The Nyquist plots of GCE, p-GCE, and VMSF/p-GCE were measured under open-circuit voltage to further study the electrochemical behaviors of different electrodes (Figure 2B). The insets are the schematic illustration of the equivalent circuit (left inset) and the enlarged view of curves in a high-frequency region (right inset). The equivalent circuit contains the solution resistance (*R*_s_), double layer capacitance (*C*_dl_), Warburg impedance (*Z*_w_), and apparent charge transfer resistance (*R*_ct_). As shown, each electrode exhibits a semicircle in the high-frequency region representing the electron transfer–limiting process and a linear portion at the low-frequency region corresponding to the diffusion-limited process. As known, the effective diameter of the semicircle in a high-frequency region is equal to *R*_ct_ which is responsible for electron transfer kinetics of redox reactions at the electrode–electrolyte interface. Supplementary Table S2 displays the *R*_s_ and *R*_ct_ obtained on different electrodes. As shown, p-GCE shows the lowest *R*_ct_ (61 Ω), suggesting a faster charge transfer kinetics at the electrode interface after electrochemical pretreatment (the *R*_ct_ of GCE is 82 Ω). After p-GCE is covered by SM-blocked nanochannels, a remarkably increased *R*_ct_ (407 Ω) is observed on SM@VMSF/p-GCE owing to the inhibited probe diffusion. When the SM is removed, the *R*_ct_ (302 Ω) of VMSF/p-GCE further decreases owing to the permeability of the high-density nanochannels of the VMSF. However, it is still higher than that of p-GCE, which is due to the electrostatic repulsion between negatively charged VMSF^−^ and Fe(CN)_6_^3-/4^.

As demonstrated by transmission electron microscopy (TEM) images, the VMSF possesses homogenously distributed nanopores with a hexagonally packed structure and uniform diameter between 2 and 3 nm (Figure 2C). The porosity is ∼45%. The cross-sectional TEM image proves the perpendicular orientation of nanochannels to the substrate (Figure 2D).

### Enhanced Electrochemical Response of BPA on VMSF/p-GCE

**FIGURE 3:**
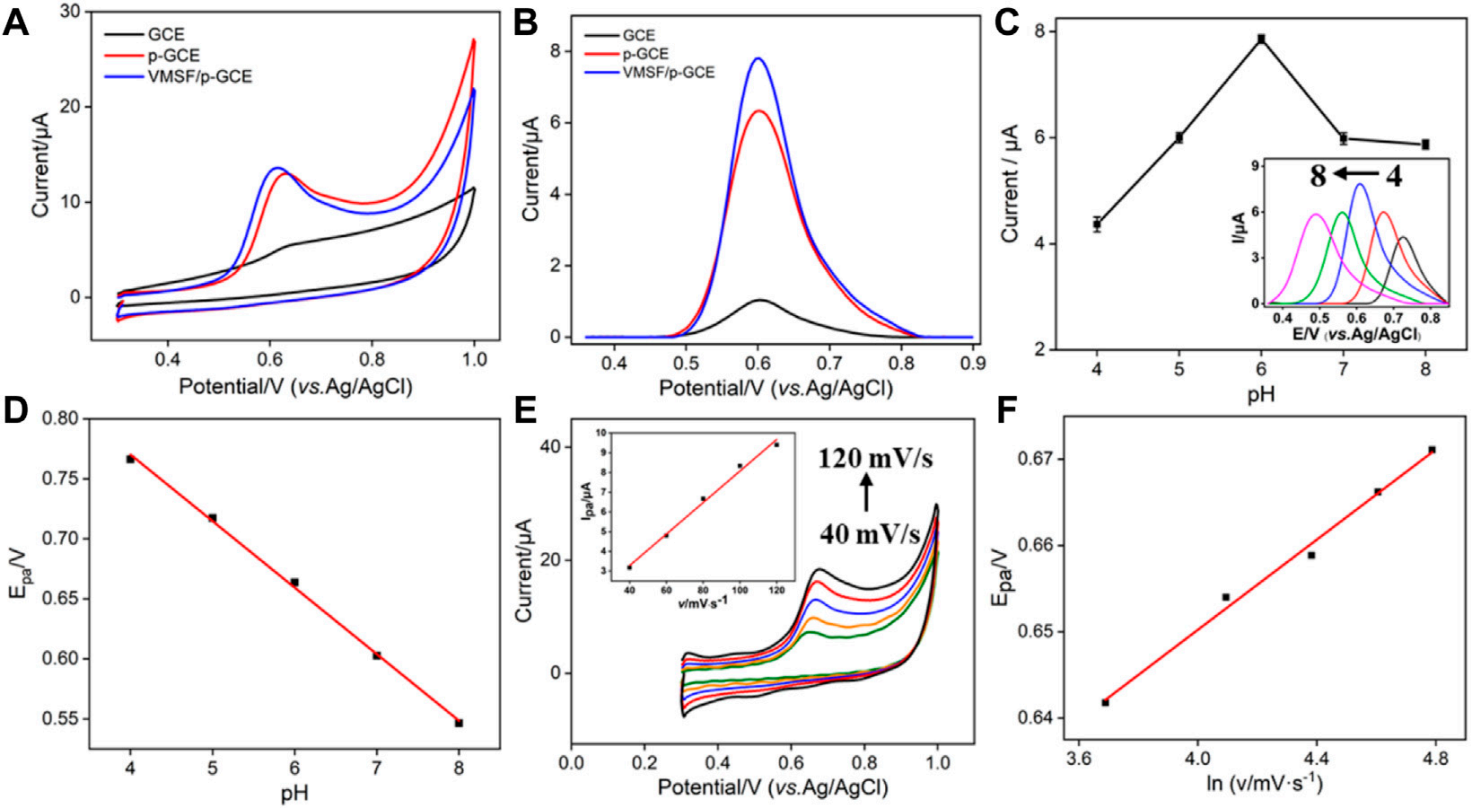
CV **(A)** and DPV **(B)** curves obtained on GCE, p-GCE, and VMSF/p-GCE in PBS (0.1 M, pH = 6) containing BPA (10 μM). The dependence of DPV anodic peak current **(C)** and CV anodic peak potential **(D)** on the pH value. Inset in c is the corresponding DPV curves in BPA (10 μM). **(E)** CV curves of BPA (10 μM) on the VMSF/p-GCE with different scan rates (40, 70, 100, 130, 160, 190, and 220 mV/s). The inset is the plot of the scan rate *vs.* peak current. **(F)** Dependence of *E*_pa_ on the natural logarithm of scan rate.

The electrochemical behavior of BPA at the VMSF/p-GCE, p-GCE and GCE was investigated. As shown in Figure 3A, BPA shows an irreversible oxidation process at these three types of electrodes, which is consistent with many previous reports. (Pang et al., 2020; Freitas et al., 2020; Hu et al., 2016) In comparison with the GCE, p-GCE exhibits a rather higher peak current. This is mainly ascribed to the increase of the electroactive surface and electrocatalytic ability through electrochemical activation (Figure 3B). (Xuan et al., 2021) Owing to the interaction between the abundant silanol groups on the nanochannel surface and phenolic hydroxyl groups on BPA through hydrogen bond, the oxidation current of BPA further increases after equipment of the VMSF on p-GCE (Figure 3B). Due to the enrichment effect from both the supporting electrode and nanochannels, the VMSF/p-GCE can realize dual-signal amplification, leading to high sensitivity in detection.

### Optimized Conditions for BPA Detection

To achieve an excellent performance toward the analysis of BPA, the detection conditions were optimized. As shown in Figure 3C, the peak current first increases with the increase of pH and reaches the highest value at pH 6. Then, the peak current decreases with the increase of pH. The decrease of the peak current at higher pH values might be attributed to the electrostatic repulsion of the negatively charged VMSF toward anionic BPA (p*K*_a1_ = 9.6, p*K*_a2_ = 10.2). The decline of peak current in an acid medium might result from the present H^+^ ion, which is one of the products of electro-oxidation of BPA, posing an inhibiting effect on the oxidation peak current. In addition, *E*_pa_ shifts negatively in the investigated pH range (Figure 3D). A good linear relationship is revealed between *E*_pa_ and the pH value (*E*_pa_ = −0.055 pH + 0.9919, *R* ^2^ = 0.9980). The number of protons and electrons involved in the oxidation of BPA is calculated using the following equation (Eq. 1):

$$\frac{dE_{pa}}{dpH}=2.303\frac{mRT}{nF},$$ (1)

where *R* is the gas constant (*R* = 8.314 J mol^−1^ K^−1^), *T* is the absolute temperature (*T* = 298 K), *F* is the Faraday constant (*F* = 96,485 C mol^−1^), and *m* and *n* are the number of protons and electrons, respectively. The ratio of *m/n* is calculated to be 0.93, indicating that the number of protons and electrons involved in the oxidation of BPA is same. To further study the mechanism of the electro-oxidation process of BPA at VMSF/p-GCE, the electrochemical signal of BPA at different scan rates is investigated (Figure 3E). A linear relationship between peak current (*I*) and scan rate (*v*) is found (*I* = 0.0799*v* + 0.0855, *R* ^2^ = 0.9899), indicating that the electro-oxidation of BPA at the VMSF/p-GCE is adsorption-controlled. The relationship between peak potential (*E*_pa_) and scan rate (*v*) in a completely irreversible process can be described according to the following Laviron equation (Eq. 2):

$$E_{pa}=E^{o}+\left(\frac{RT}{\alpha nF}\right)\ln\frac{RTK_{s}}{\alpha nF}+\left(\frac{RT}{\alpha nF}\right)\ln v,$$ (2)

where *E* ^o^ is the formal redox potential; *α* is the transfer coefficient, which is assumed to be 0.5 in a total irreversible electrochemical reaction; *n* is the number of electron transfers involved in the rate-determining step; and *k*_s_ is the standard rate constant of the reaction. The other quantities are the same as mentioned earlier. Accordingly, the relationship between *E*_pa_ and *v* can be depicted as *E*_pa_ = 0.0261l n *v* + 0.0546 (*R* ^2^ = 0.9905, Figure 3F). The value of *n* is calculated as 1.97. Therefore, the oxidation of BPA on the VMSF/p-GCE is a 2H^+^/2e^−^ transfer process, which can be described as the following equation (Eq. 3).

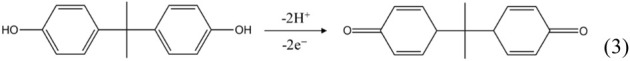

3



**FIGURE 4:**
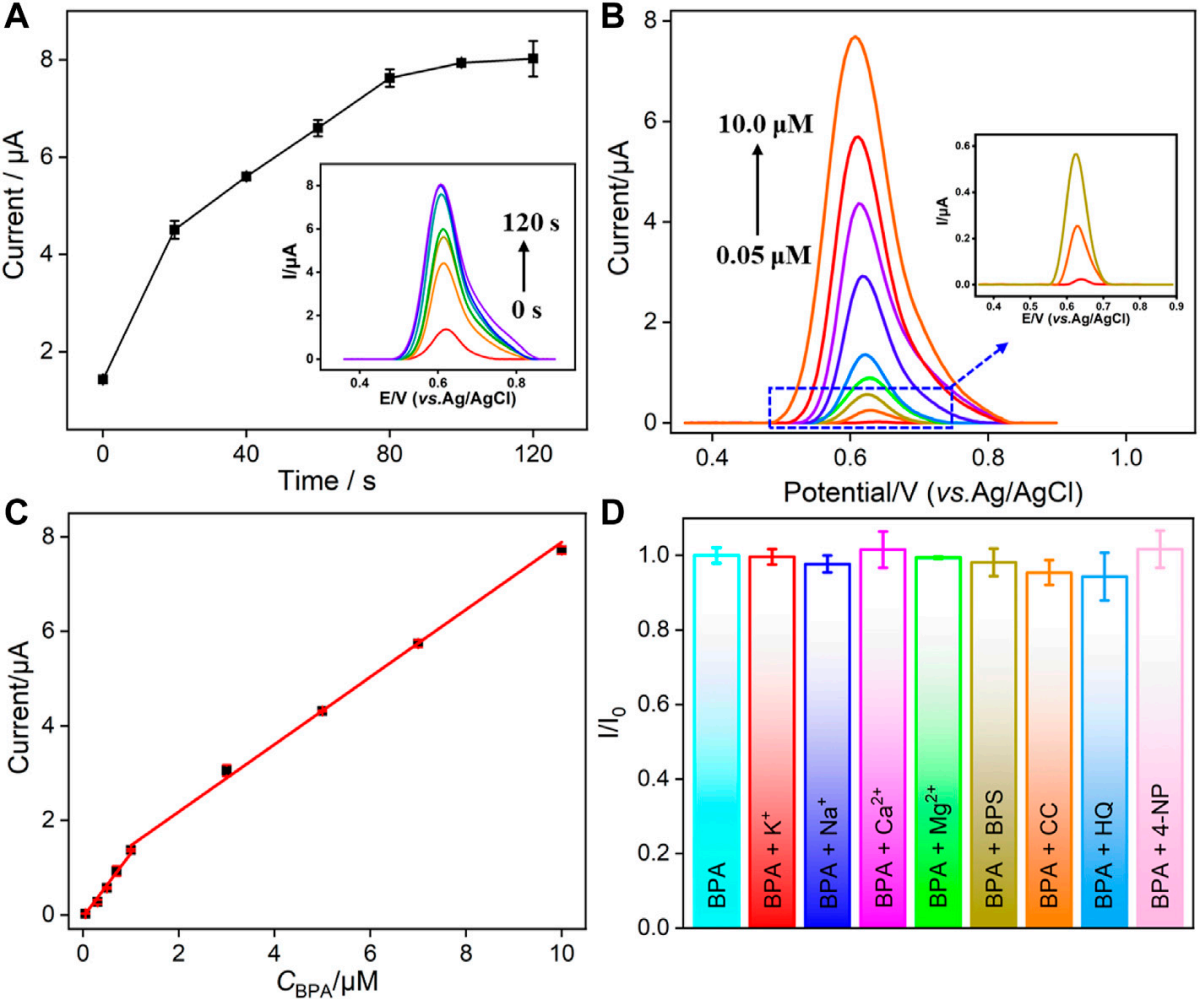
**(A)** Dependence of the stirring time on the current response of BPA on VMSF/p-GCE. Inset is the corresponding DPV curves. **(B)** DPV curves of VMSF/p-GCE obtained in PBS (0.1 M, pH = 6) containing different concentrations of BPA after stirring for 100 s. Inset is the magnified view of the DPV curves in the low-concentration region. **(C)** Calibration curve for BPA. **(D)** The current ratio (*I*/*I*_0_) obtained from VMSF/p-GCE for the detection of BPA (5.0 μM) in the absence (*I*_0_) and presence (*I*) of 5-fold (BPS and 4-NP) or 50-fold of other added interfering species. The error bars represent the standard deviation (SD) of three measurements.

Since the oxidation of BPA on VMSF/p-GCE is adsorption-controlled as mentioned previously, the accumulation time before detection is further optimized. As shown in Figure 4A, the oxidation peak current increases when the stirring time is increased and then reaches a platform at 100 s. Thus, an accumulation time of 100 s is chosen for further investigation.

### Voltammetric Determination of BPA Using the VMSF/p-GCE

Figure 4B shows the differential pulse voltammetry (DPV) curves obtained on the VMSF/p-GCE in the presence of different concentrations of BPA. A good linear correlation was found between the peak current (*I*) and the concentration of BPA (*C*_BPA_) from 50 nM to 1.0 μM (*I* = 1.348*C*_BPA_ − 0.0425, *R* ^2^ = 0.9915) and 1.0–10.0 μM (*I* = 0.7571*C*_BPA_ + 0.7130, *R* ^2^ = 0.9938). The limit of detection (LOD) is calculated to be as low as 15 nM, at a signal-to-noise ratio of 3(S/N = 3). The comparison between the determination of BPA using different electrodes is demonstrated in Table 1. The LOD is lower than that obtained from graphene oxide-poly (1-[3-(N-pyrrolyl) propyl]-3-butylimidazolium bromide)–modified GCE (GO-poly (NPBimBr)/GCE) (Wang et al., 2018), tyrosinase-graphdiyne-chitosan–modified GCE (Tyr-GDY-CS/GCE) (Wu et al., 2020), Ag nanoparticle/multiwalled carbon nanotube–modified GCE (AgNP/MWCNT/GCE) (Goulart et al., 2018), reduced graphene oxide-Fe_3_O_4_/chitosan/laccase–modified GCE (rGO-Fe_3_O_4_/CS/laccase/GCE) (Fernandes et al., 2020), and nanoporous PtFe/graphene–modified GCE (NP-PtFe/Gr/GCE) (Tian et al., 2018) but higher than that obtained on Cu_2_O-CuO@graphene quantum dot–modified GCE (Cu_2_O-CuO@GQD/GCE) (Ashraf et al., 2020), GO-MWCNT-*β*-cyclodextrin–modified screen-printed carbon electrode (GO-MWCNT-*β*CD/SPE) (Alam and Deen, 2020), molecularly imprinted polymer/polypyrole-modified laser-scribed graphene (MIP/PPy@LSG) (Beduk et al., 2020), and NiS/rGO-modified mechanical pencil lead (NiS/rGO/MPL). (Vu et al., 2019).

**TABLE 1:** Comparison between electrochemical detection of BPA using a different electrode.

Electrode materials	Method	Detection range (μM)	LOD (nM)	Ref
VMSF/p-GCE	DPV	0.05–10.0	15	This work
Cu_2_O-CuO@GQD/GCE	CA	0.002–10000	1	29
GO-MWCNT-βCD/SPE	LSV	0.05–30	6	32
GO-poly(NPBimBr)/GCE	DPV	0.2–10.0	17	36
MIP/PPy@LSG	DPV	0.05–5.0	8	38
Tyr-GDY-CS/GCE	CA	0.1–3.5	24	61
AgNP/MWCNT/GCE	SWV	5.0–152	2,400	62
rGO-Fe_3_O_4_/CS/laccase/GCE	SWV	0.025–20	47	63
NP-PtFe/Gr/GCE	DPV	0.2–96	170	64
NiS/rGO/MPL	ASV	0.043–0.26	1.75	65

GCE, glassy carbon electrode; GQDs, graphene quantum dots; GO, graphene oxide; MWCNT, multiwalled carbon nanotube; *β*CD, *β*-cyclodextrin; SPE, screen-printed carbon electrode; NPBimBr, 1-[3-(N-pyrrolyl) propyl]-3-butylimidazolium bromide; MIP, molecularly imprinted polymer; PPy, polypyrole; LSG, laser-scribed graphene; Tyr, tyrosinase; GDY, graphdiyne; CS, chitosan; AgNP, Ag nanoparticle; rGO, reduced graphene oxide; NP, nanoporous; Gr, graphene; MPL, mechanical pencil lead; DPV, differential pulse voltammetry; LSV, linear sweep voltammetry; CA, chronoamperometry; SWV, square wave voltammetry; ASV, anodic stripping voltammetry.

### Selectivity and Anti-Interference Ability of VMSF/p-GCE

**FIGURE 5:**
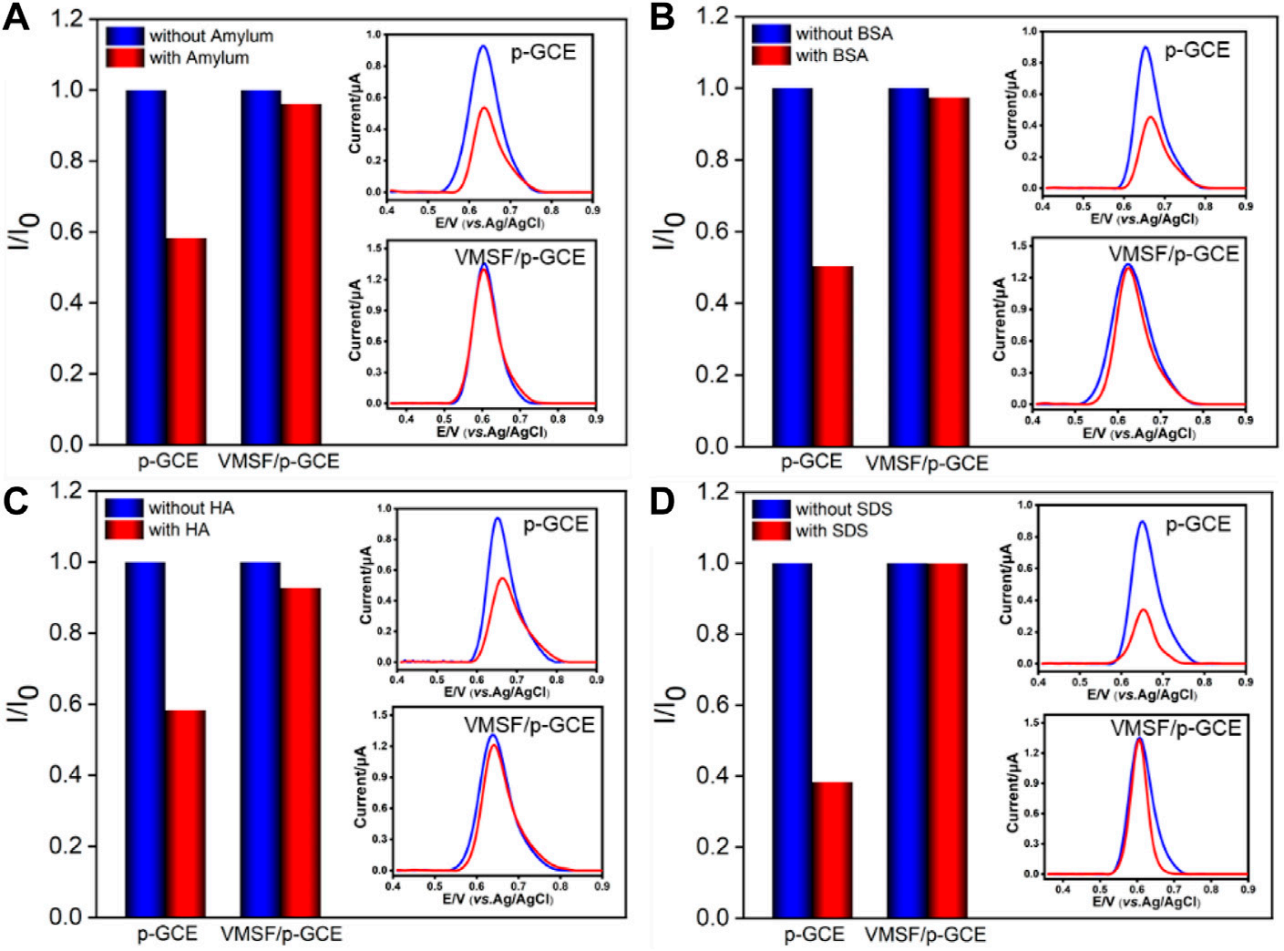
Normalized oxidation peak current ratio on p-GCE and VMSF/p-GCE toward BPA (1.0 μM). *I* and *I*_0_ represent the currents obtained in the presence and absence of 50 μg/ml of amylum **(A)**, BSA **(B)**, HA **(C)**, or SDS **(D)** in PBS (0.1 M, pH = 6). The insets are the corresponding DPV curves obtained on p-GCE and VMSF/p-GCE in the absence and presence of the fouling species.

The detection selectivity is critical for the real application of the electrochemical sensor. To evaluate the selectivity, the performance of the developed VMSF/p-GCE sensor to detect BPA in the presence of common metal ions (K^+^, Na^+^, Ca^2+^, and Mg^2+^) and electroactive environmental pollutants (BPS, CC, HQ, and 4-NP) is investigated. As shown in Figure 4D, these co-existed molecules do not interfere with the detection of BPA. The high selectivity is attributed to the good potential resolution ability of p-GCE. On the one hand, the co-existed ions cannot be oxidated in the applied potential range. On the other hand, electroactive organic molecules have different electrochemical behaviors and different electrocatalytic oxidation potentials on p-GCE owing to its good electrocatalytic activity. Thus, the detection of BPA has good selectivity. In addition, polysaccharides (starch), proteins (BSA), organic macromolecules (humic acid, HA), and surfactants (sodium dodecyl sulfate, SDS) that usually exist in complex environmental samples are selected as model substances to evaluate the anti-interference ability of the VMSF/p-GCE sensor in complex matrices. Figure 5 shows the ratio of current responses (*I*/*I*_0_) to BPA before (*I*_0_) and after (*I*) incubation of p-GCE or the VMSF/p-GCE with one of the possible interferences for 10 min. As seen, the current signals of p-GCE significantly decrease by nearly 50% in the presence of the interferences, indicating a severe matrix effect. Thus, the substances that are frequently present in complex samples can remarkably passivate the electrode, leading to a serious change of sensitivity. In contrast, almost no significant current change is observed for the VMSF/p-GCE, suggesting an excellent anti-smudge ability. This is attributed to the fact that the nanochannels of the VMSF can protect the electrodes from severe contamination in complex matrices through size repulsion and charge repulsion effects, which endow the VMSF-modified electrodes with a good antifouling ability. Thus, the VMSF/p-GCE sensor has great potential for the direct detection of complex samples. In comparison with other optical or chromatographic strategies (e.g. analysis), electroanalysis has advantages of simple instruments, fast detection, and high selectivity. (Cui et al., 2020; Zhao et al., 2020).

### Repeatability, Reproducibility, and Stability of the VMSF/p-GCE

**FIGURE 6:**
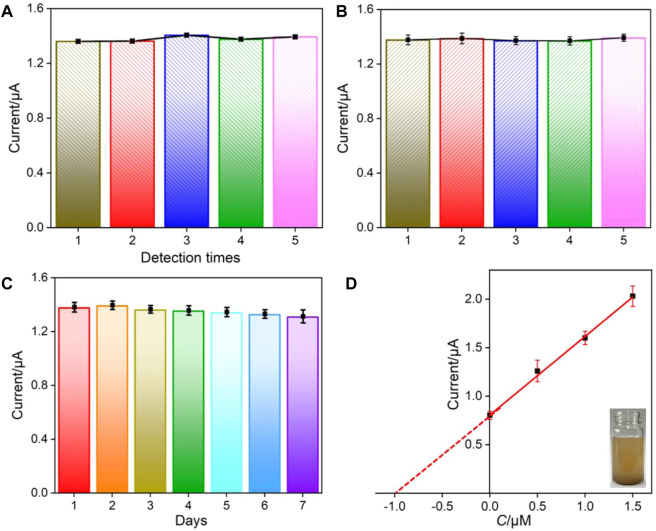
Repeatability **(A)**, reproducibility **(B)**, and stability **(C)** of VMSF/p-GCE for the detection of BPA (1.0 μM). **(D)** Linear relationship of the DPV current *vs*. the concentration of spiked BPA in SLS. Inset is the digital picture of the detected SLS. The error bars represent the standard deviation (SD) of the three measurements.

The repeatability, reproducibility, and stability of the developed VMSF/p-GCE sensor are also examined (Figures 6A–C). The repeatability was evaluated by detecting BPA (1.0 μM) five times using the same electrode. The electrode is easily regenerated by immersing in an HCl–ethanol (0.1 M) solution for 5 min. A relative standard deviation (RSD) of 1.4% is found, suggesting satisfactory repeatability (Figure 6A). To investigate the reproducibility of the sensor, five electrodes are parallelly fabricated under the same conditions. A low RSD of 0.7% is revealed for detecting BPA (1.0 μM), indicating high reproducibility (Figure 6B). The stability of the developed sensor is investigated by comparing the detection of BPA (1.0 μM) before and after storage in 4°C for 7 days. As shown in Figure 6C, the peak current is 95.0% that of the freshly fabricated electrode, confirming high stability of the fabricated sensor.

### Direct Detection of BPA in Environment Samples

Considering the excellent antifouling ability of the VMSF/p-GCE sensor, the direct analysis of environmental water and soil leaching solutions is investigated using a standard addition method. As shown in Table 2, the detection exhibits satisfactory recoveries ranged from 98.0–104.5%, with a low relative standard deviation (RSD≤ 3.9%), indicating high reliability. As demonstrated in the inset of Figure 6D, the analyzed soil leaching solution is a suspension. However, the detected concentration of artificially added BPA (0.99 μM) by the extrapolation method (*I* = 0.810*C* + 0.806, *R* ^2^ = 0.9978) is quite similar with the added concentration (1.0 μM). These prove the reliability of the VMSF/p-GCE for direct BPA analysis in real complex samples without separation.

**TABLE 2:** Detection of BPA in environmental water samples.

Sample	Added/μM	Found/μM	RSD/% (*n* = 3)	Recovery/%
Pond water^a^	0.50	0.492	3.9	98.0
	2.00	2.09	3.7	104.5
	5.00	4.95	3.8	99.0
Lake water^b^	0.10	0.103	1.8	104.2
	3.00	3.14	3.9	103.5
	6.00	5.86	1.3	97.9

a diluted with PBS (0.1 M, pH = 6) for 10 times.

b diluted with PBS (0.1 M, pH = 6) for 10 times.

## Conclusion

In summary, we have developed an electrochemical sensing platform based on the equipment of the VMSF on p-GCE for direct and sensitive analyses of BPA in complex environmental samples. The supporting p-GCE offers a high electrode surface area, excellent electrocatalytic performance, and good potential resolution ability. In addition, the nanochannels of the VMSF act as nanofilter to endow the modified electrode with an anti-interference ability. On the other hand, nanochannels can also enrich analytes through electrostatic or hydrogen bonding to realize signal amplification. Without separation, rapid and sensitive detection of BPA in pond or lake water and soil leaching solutions is realized with high reliability. In comparison with the developed method for the detection of BPA, the developed VMSF/p-GCE sensor has simple electrode structure and excellent sensing performance. In combination with the further modification of the supporting electrode or the VMSF nanochannel, the sensor demonstrated here may be extended for detecting a variety of analytes in complex samples in medical, biological, food, environmental, and other fields.

## Data Availability

The original contributions presented in the study are included in the article/Supplementary Material, further inquiries can be directed to the corresponding authors.

## References

[B1] Alam A. U. Deen M. J. (2020). Bisphenol A Electrochemical Sensor Using Graphene Oxide and β-Cyclodextrin-Functionalized Multi-Walled Carbon Nanotubes. Anal. Chem. 92, 5532–5539. 10.1021/acs.analchem.0c00402 32141295

[B2] Ashraf G. Asif M. Aziz A. Dao A. Q. Zhang T. Iftikhar T. (2020). Facet-energy Inspired Metal Oxide Extended Hexapods Decorated with Graphene Quantum Dots: Sensitive Detection of Bisphenol A in Live Cells. Nanoscale 12, 9014–9023. 10.1039/c9nr10944g 32270807

[B3] Authority E. F. S. (2006). Opinion of the Scientific Panel on Food Additives, Flavourings, Processing Aids and Materials in Contact with Food on a Request from the Commission Related to BPA, 1–75.

[B4] Beduk T. Ait Lahcen A. Tashkandi N. Salama K. N. (2020). One-step Electrosynthesized Molecularly Imprinted Polymer on Laser Scribed Graphene Bisphenol A Sensor. Sensors Actuators B Chem. 314, 128026–128035. 10.1016/j.snb.2020.128026

[B5] Bilal M. Iqbal H. M. N. Barceló D. (2019). Mitigation of Bisphenol A Using an Array of Laccase-Based Robust Bio-Catalytic Cues - a Review. Sci. Total Environ. 689, 160–177. 10.1016/j.scitotenv.2019.06.403 31271985

[B6] Chung E. Jeon J. Yu J. Lee C. Choo J. (2015). Surface-enhanced Raman Scattering Aptasensor for Ultrasensitive Trace Analysis of Bisphenol A. Biosens. Bioelectron. 64, 560–565. 10.1016/j.bios.2014.09.087 25310489

[B7] Correia-Sá L. Norberto S. Delerue-Matos C. Calhau C. Domingues V. F. (2018). Micro-QuEChERS Extraction Coupled to GC-MS for a Fast Determination of Bisphenol A in Human Urine. J. Chromatogr. B 1072, 9–16. 10.1016/j.jchromb.2017.10.060 29132025

[B8] Cui Y. Duan W. Jin Y. Wo F. Xi F. Wu J. (2020). Ratiometric Fluorescent Nanohybrid for Noninvasive and Visual Monitoring of Sweat Glucose. ACS Sens. 5, 2096–2105. 10.1021/acssensors.0c00718 32450686

[B9] Cui Y. Duan W. Jin Y. Wo F. Xi F. Wu J. (2021). Graphene Quantum Dot-Decorated Luminescent Porous Silicon Dressing for Theranostics of Diabetic Wounds. Acta Biomater. 131, 544–554. 10.1016/j.actbio.2021.07.018 34265475

[B10] Deng X. Zhao J. Ding Y. Tang H. Xi F. (2021). Iron and Nitrogen Co-doped Graphene Quantum Dots as Highly Active Peroxidases for the Sensitive Detection of L-Cysteine. New J. Chem. 45, 19056–19064. 10.1039/d1nj03559b

[B11] Ding H. Guo W. Zhou P. Su B. (2020). Nanocage-confined Electrochemiluminescence for the Detection of Dopamine Released from Living Cells. Chem. Commun. 56, 8249–8252. 10.1039/d0cc03370g 32558869

[B12] Duan W. Jin Y. Cui Y. Xi F. Liu X. Wo F. (2021). A Co-delivery Platform for Synergistic Promotion of Angiogenesis Based on Biodegradable, Therapeutic and Self-Reporting Luminescent Porous Silicon Microparticles. Biomaterials 272, 120772–120784. 10.1016/j.biomaterials.2021.120772 33838529

[B13] Engstrom R. C. Strasser V. A. (1984). Characterization of Electrochemically Pretreated Glassy Carbon Electrodes. Anal. Chem. 56, 136–141. 10.1021/ac00266a005

[B14] Fernandes P. M. V. Campiña J. M. Silva A. F. (2020). A Layered Nanocomposite of Laccase, Chitosan, and Fe3O4 Nanoparticles-Reduced Graphene Oxide for the Nanomolar Electrochemical Detection of Bisphenol A. Microchim. Acta 187, 262–271. 10.1007/s00604-020-4223-x 32270383

[B15] Freitas J. M. Wachter N. Rocha-Filho R. C. (2020). Determination of Bisphenol S, Simultaneously to Bisphenol A in Different Water Matrices or Solely in Electrolyzed Solutions, Using a Cathodically Pretreated Boron-Doped Diamond Electrode. Talanta 217, 121041–121049. 10.1016/j.talanta.2020.121041 32498895

[B16] Ghalkhani M. Sohouli E. (2022). Synthesis of the Decorated Carbon Nano Onions with Aminated MCM-41/Fe3O4 NPs: Morphology and Electrochemical Sensing Performance for Methotrexate Analysis. Microporous Mesoporous Mater. 331, 111658–111667. 10.1016/j.micromeso.2021.111658

[B17] Goulart L. A. Gonçalves R. Correa A. A. Pereira E. C. Mascaro L. H. (2018). Synergic Effect of Silver Nanoparticles and Carbon Nanotubes on the Simultaneous Voltammetric Determination of Hydroquinone, Catechol, Bisphenol A and Phenol. Microchim. Acta 185, 12–20. 10.1007/s00604-017-2540-5 29594601

[B18] Hadi M. Cheng C. Wu J. Chen J. Shigotoshi E. Esmaeil Najafi A. (2016). A Highly Sensitive and Specific Capacitive Aptasensor for Rapid and Label-free Trace Analysis of Bisphenol A (BPA) in Canned Foods. Biosens. Bioelectron. 89, 1059–1067. 10.1016/j.bios.2016.09.109 27825518

[B19] Herzog G. Sibottier E. Etienne M. Walcarius A. (2013). Electrochemically Assisted Self-Assembly of Ordered and Functionalized Mesoporous Silica Films: Impact of the Electrode Geometry and Size on Film Formation and Properties. Faraday Discuss. 164, 259–273. 10.1039/c3fd00021d 24466668

[B20] Hu L. Fong C.-C. Zhang X. Chan L. L. Lam P. K. S. Chu P. K. (2016). Au Nanoparticles Decorated TiO2 Nanotube Arrays as a Recyclable Sensor for Photoenhanced Electrochemical Detection of Bisphenol A. Environ. Sci. Technol. 50, 4430–4438. 10.1021/acs.est.5b05857 27002339

[B21] Jun Y. Yang L. Ming X. Qing Y. Tongyi L. (2020). The Electrochemical Behaviors and Kinetics of AuNPs/N, S-GQDs Composite Electrode: a Novel Label-free Amplified BPA Aptasensor with Extreme Sensitivity and Selectivity. J. Mol. Liq. 320, 114384–114392. 10.1016/j.molliq.2020.114384

[B22] Kumar Naik T. S. S. Anil A. G. Swamy B. E. K. Singh S. Madhavi V. Raghavendra S. M. (2022). A Novel Electrochemical Sensor Based on 2,6-bis (2-benzimidazoyl) Pyridine for the Detection of Bisphenol A. Mater. Chem. Phys. 275, 125287. 10.1016/j.matchemphys.2021.125287

[B23] Lee E.-H. Lim H. J. Lee S.-D. Son A. (2017). Highly Sensitive Detection of Bisphenol A by Nanoaptamer Assay with Truncated Aptamer. ACS Appl. Mat. Interfaces 9, 14889–14898. 10.1021/acsami.7b02377 28393521

[B24] Lee E.-H. Lee S. K. Kim M. J. Lee S.-W. (2019). Simple and Rapid Detection of Bisphenol A Using a Gold Nanoparticle-Based Colorimetric Aptasensor. Food Chem. 287, 205–213. 10.1016/j.foodchem.2019.02.079 30857691

[B25] Lei Y. Zhang Y. Wang B. Zhang Z. Yuan L. Li J. (2022). A Lab-On-Injector Device with Au Nanodots Confined in Carbon Nanofibers for *In Situ* Electrochemical BPA Sensing in Beverages. Food control. 134, 108747–108756. 10.1016/j.foodcont.2021.108747

[B26] Li Y. Wang H. Yan B. Zhang H. (2017). An Electrochemical Sensor for the Determination of Bisphenol A Using Glassy Carbon Electrode Modified with Reduced Graphene Oxide-Silver/poly-L-Lysine Nanocomposites. J. Electroanal. Chem. 805, 39–46. 10.1016/j.jelechem.2017.10.022

[B27] Li S. Zhang D. Liu J. Cheng C. Zhu L. Li C. (2019). Electrochemiluminescence on Smartphone with Silica Nanopores Membrane Modified Electrodes for Nitroaromatic Explosives Detection. Biosens. Bioelectron. 129, 284–291. 10.1016/j.bios.2018.09.055 30245166

[B28] Li Y. Zhou J. Song J. Liang X. Zhang Z. Men D. (2019). Chemical Nature of Electrochemical Activation of Carbon Electrodes. Biosens. Bioelectron. 144, 111534–111541. 10.1016/j.bios.2019.111534 31518791

[B29] Li D. Li C. Wang H. Li J. Zhao Y. Jiang X. (2021). Single-atom Fe Catalytic Amplification-Gold Nanosol SERS/RRS Aptamer as Platform for the Quantification of Trace Pollutants. Microchim. Acta 188, 175–185. 10.1007/s00604-021-04828-8 33893886

[B30] Liu Q. Zhong H. Chen M. Zhao C. Liu Y. Xi F. (2020). Functional Nanostructure-Loaded Three-Dimensional Graphene Foam as a Non-enzymatic Electrochemical Sensor for Reagentless Glucose Detection. RSC Adv. 10, 33739–33746. 10.1039/d0ra05553k 35519067 PMC9056722

[B31] Ma X. Liao W. Zhou H. Tong Y. Yan F. Tang H. (2020). Highly Sensitive Detection of Rutin in Pharmaceuticals and Human Serum Using ITO Electrodes Modified with Vertically-Ordered Mesoporous Silica-Graphene Nanocomposite Films. J. Mat. Chem. B 8, 10630–10636. 10.1039/d0tb01996h 33146656

[B32] Md Younus A. Arif Ul A. Matiar M. R. H. (2020). Fabrication of Highly Sensitive Bisphenol A Electrochemical Sensor Amplified with Chemically Modified Multiwall Carbon Nanotubes and *β*-cyclodextrin. Sens. Actuators B Chem. 320, 128319–128328.

[B33] Nasir T. Zhang L. Vilà N. Herzog G. Walcarius A. (2016). Electrografting of 3-aminopropyltriethoxysilane on a Glassy Carbon Electrode for the Improved Adhesion of Vertically Oriented Mesoporous Silica Thin Films. Langmuir 32, 4323–4332. 10.1021/acs.langmuir.6b00798 27065214

[B34] Nasir T. Herzog G. Hébrant M. Despas C. Liu L. Walcarius A. (2018). Mesoporous Silica Thin Films for Improved Electrochemical Detection of Paraquat. ACS Sens. 3, 484–493. 10.1021/acssensors.7b00920 29338195

[B35] Pang Y.-H. Huang Y.-Y. Wang L. Shen X.-F. Wang Y.-Y. (2020). Determination of Bisphenol A and Bisphenol S by a Covalent Organic Framework Electrochemical Sensor. Environ. Pollut. 263, 114616–114626. 10.1016/j.envpol.2020.114616

[B36] Ponnada S. Gorle D. B. Kiai M. S. Raju C. V. Faraji M. Sharma R. K. (2022). Understanding the Endocrine Disruptor and Determination of Bisphenol A by Functional Cu-BTABB-MOF/rGO Composite as Facile Rapid Electrochemical Sensor: an Experimental and DFT Investigation. Anal. Methods 14, 560–573. 10.1039/d1ay02150h 35050283

[B37] Rajendran J. Kannan T. S. Dhanasekaran L. S. Murugan P. Atchudan R. Alothman Z. A. (2022). Preparation of 2D Graphene/MXene Nanocomposite for the Electrochemical Determination of Hazardous Bisphenol A in Plastic Products. Chemosphere 287, 132106–132115. 10.1016/j.chemosphere.2021.132106 34507149

[B38] Sabbaghan M. Ghalkhani M. Hosseini M. Ghanbari M. (2021). Mn-doped ZnS Synthesis in DABCO Based Ionic Liquid: Morphology and Electrochemical Sensing Performance for Isoprenaline Analysis. J. Industrial Eng. Chem. 95, 367–375. 10.1016/j.jiec.2021.01.012

[B39] Shi K. Shiu K.-K. (2002). Scanning Tunneling Microscopic and Voltammetric Studies of the Surface Structures of an Electrochemically Activated Glassy Carbon Electrode. Anal. Chem. 74, 879–885. 10.1021/ac010734+ 11866068

[B40] Takayanagi S. Tokunaga T. Liu X. Okada H. Matsushima A. Shimohigashi Y. (2006). Endocrine Disruptor Bisphenol A Strongly Binds to Human Estrogen-Related Receptor γ (ERRγ) with High Constitutive Activity. Toxicol. Lett. 167, 95–105. 10.1016/j.toxlet.2006.08.012 17049190

[B41] Tian C. Chen D. Lu N. Li Y. Cui R. Han Z. (2018). Electrochemical Bisphenol A Sensor Based on Nanoporous PtFe Alloy and Graphene Modified Glassy Carbon Electrode. J. Electroanal. Chem. 830-831, 27–33. 10.1016/j.jelechem.2018.10.023

[B42] Vu T. D. Khac Duy P. Bui H. T. Han S.-H. Chung H. (2019). Reduced Graphene Oxide-Nickel Sulfide (NiS) Composited on Mechanical Pencil Lead as a Versatile and Cost-Effective Sensor for Electrochemical Measurements of Bisphenol A and Mercury (II). Sensors Actuators B Chem. 281, 320–325. 10.1016/j.snb.2018.08.139

[B43] Walcarius A. Sibottier E. Etienne M. Ghanbaja J. (2007). Electrochemically Assisted Self-Assembly of Mesoporous Silica Thin Films. Nat. Mater 6, 602–608. 10.1038/nmat1951 17589513

[B44] Wan Y. Zhao J. Deng X. Chen J. Xi F. Wang X. (2021). Colorimetric and Fluorescent Dual-Modality Sensing Platform Based on Fluorescent Nanozyme. Front. Chem. 9, 774486–774497. 10.3389/fchem.2021.774486 34869222 PMC8635524

[B45] Wang Y. Li C. Wu T. Ye X. (2018). Polymerized Ionic Liquid Functionalized Graphene Oxide Nanosheets as a Sensitive Platform for Bisphenol A Sensing. Carbon 129, 21–28. 10.1016/j.carbon.2017.11.090

[B46] Wang Y. Zhao X. Huo B. Ren S. Bai J. Peng Y. (2020). Sensitive Fluorescence Aptasensor Based on Hybridization Chain Reaction with Upconversion Nanoparticles by Triplex DNA Formation for Bisphenol A Detection. ACS Appl. Bio Mat. 4, 763–769. 10.1021/acsabm.0c01347

[B47] Wang Y. Yin C. Zhuang Q. (2020). An Electrochemical Sensor Modified with Nickel Nanoparticle/nitrogen-Doped Carbon Nanosheet Nanocomposite for Bisphenol A Detection. J. Alloys Compd. 827, 154335–154345. 10.1016/j.jallcom.2020.154335

[B48] Wang H. Liu Z.-h. Tang Z. Zhang J. Dang Z. Liu Y. (2021). Possible Overestimation of Bisphenol Analogues in Municipal Wastewater Analyzed with GC-MS. Environ. Pollut. 273, 116505–116511. 10.1016/j.envpol.2021.116505 33484998

[B49] Wang Y. Liang Y. Zhang S. Wang T. Zhuang X. Tian C. (2021). Enhanced Electrochemical Sensor Based on Gold Nanoparticles and MoS2 Nanoflowers Decorated Ionic Liquid-Functionalized Graphene for Sensitive Detection of Bisphenol A in Environmental Water. Microchem. J. 161, 105769–105776. 10.1016/j.microc.2020.105769

[B50] Wang M. Lin J. Gong J. Ma M. Tang H. Liu J. (2021). Rapid and Sensitive Determination of Doxorubicin in Human Whole Blood by Vertically-Ordered Mesoporous Silica Film Modified Electrochemically Pretreated Glassy Carbon Electrodes. RSC Adv. 11, 9021–9028. 10.1039/d0ra10000e 35423372 PMC8695326

[B51] Wang K.-P. Hu J.-M. Zhang X. (2022). Sensitive Electrochemical Detection of Endocrine Disruptor Bisphenol A (BPA) in Milk Based on Iodine-Doped Graphene. Microchem. J. 173, 107047–107053. 10.1016/j.microc.2021.107047

[B52] Wei C. Sun S. Mandler D. Wang X. Qiao S. Z. Xu Z. J. (2019). Approaches for Measuring the Surface Areas of Metal Oxide Electrocatalysts for Determining Their Intrinsic Electrocatalytic Activity. Chem. Soc. Rev. 48, 2518–2534. 10.1039/c8cs00848e 30976760

[B53] Wetherill Y. B. Akingbemi B. T. Kanno J. McLachlan J. A. Nadal A. Sonnenschein C. (2007). *In Vitro* molecular Mechanisms of Bisphenol A Action. Reprod. Toxicol. 24, 178–198. 10.1016/j.reprotox.2007.05.010 17628395

[B54] Wu L. Gao J. Lu X. Huang C. Yi H. Chen J. (2020). Graphdiyne: a New Promising Member of 2D All-Carbon Nanomaterial as Robust Electrochemical Enzyme Biosensor Platform. Carbon 156, 568–575. 10.1016/j.carbon.2019.09.086

[B55] Xiao Y. Chen S. Zhang S. Wang G. Yi H. Xin G.-Z. (2021). Mesoporous Silica-Mediated Controllable Electrochemiluminescence Quenching for Immunosensor with Simplicity, Sensitivity and Tunable Detection Range. Talanta 231, 122399–122406. 10.1016/j.talanta.2021.122399 33965049

[B56] Xuan L. Liao W. Wang M. Zhou H. Ding Y. Yan F. (2021). Integration of Vertically-Ordered Mesoporous Silica-Nanochannel Film with Electro-Activated Glassy Carbon Electrode for Improved Electroanalysis in Complex Samples. Talanta 225, 122066–122078. 10.1016/j.talanta.2020.122066 33592785

[B57] Yan F. Chen J. Jin Q. Zhou H. Sailjoi A. Liu J. (2020). Fast One-step Fabrication of a Vertically-Ordered Mesoporous Silica-Nanochannel Film on Graphene for Direct and Sensitive Detection of Doxorubicin in Human Whole Blood. J. Mat. Chem. C 8, 7113–7119. 10.1039/d0tc00744g

[B58] Yan F. Wang M. Jin Q. Zhou H. Xie L. Tang H. (2021). Vertically-ordered Mesoporous Silica Films on Graphene for Anti-fouling Electrochemical Detection of *Tert*-Butylhydroquinone in Cosmetics and Edible Oils. J. Electroanal. Chem. 881, 114969–114975. 10.1016/j.jelechem.2020.114969

[B59] Yan F. Luo T. Jin Q. Zhou H. Sailjoi A. Dong G. (2021). Tailoring Molecular Permeability of Vertically-Ordered Mesoporous Silica-Nanochannel Films on Graphene for Selectively Enhanced Determination of Dihydroxybenzene Isomers in Environmental Water Samples. J. Hazard. Mater. 410, 124636–124644. 10.1016/j.jhazmat.2020.124636 33248825

[B60] Yang L. Chen Y. Shen Y. Yang M. Li X. Han X. (2018). SERS Strategy Based on the Modified Au Nanoparticles for Highly Sensitive Detection of Bisphenol A Residues in Milk. Talanta 179, 37–42. 10.1016/j.talanta.2017.10.055 29310247

[B61] Yang Q. Chen N. Zhang X. Ye Z. Yang Y. (2022). A Sensitive Electrochemical Sensor Based on Co_3_O_4_‐CeO_2_ Composites Modified Glassy Carbon Electrode for the Determination of Bisphenol A. ChemistrySelect 7, 1–6. 10.1002/slct.202104513

[B62] Yasri N. G. Sundramoorthy A. K. Gunasekaran S. (2015). Azo Dye Functionalized Graphene Nanoplatelets for Selective Detection of Bisphenol A and Hydrogen Peroxide. RSC Adv. 5, 87295–87305. 10.1039/c5ra16530j

[B63] Zhang J. Xu X. Chen L. (2018). An Ultrasensitive Electrochemical Bisphenol A Sensor Based on Hierarchical Ce-Metal-Organic Framework Modified with Cetyltrimethylammonium Bromide. Sensors Actuators B Chem. 261, 425–433. 10.1016/j.snb.2018.01.170

[B64] Zhang Y. Zhang W. Zhang L. Song G. Wang N. Xu W. (2021). A Molecularly Imprinted Electrochemical BPA Sensor Based on Multi-Walled Carbon Nanotubes Modified by CdTe Quantum Dots for the Detection of Bisphenol A. Microchem. J. 170, 106737–106746. 10.1016/j.microc.2021.106737

[B65] Zhao J. Zheng Y. Pang Y. Chen J. Zhang Z. Xi F. (2020). Graphene Quantum Dots as Full-Color and Stimulus Responsive Fluorescence Ink for Information Encryption. J. Colloid Interface Sci. 579, 307–314. 10.1016/j.jcis.2020.06.077 32599475

[B66] Zhou H. Ma X. Sailjoi A. Zou Y. Lin X. Yan F. (2022). Vertical Silica Nanochannels Supported by Nanocarbon Composite for Simultaneous Detection of Serotonin and Melatonin in Biological Fluids. Sensors Actuators B Chem. 353, 131101–131109. 10.1016/j.snb.2021.131101

[B67] Zhu X. Wu G. Xing Y. Wang C. Yuan X. Li B. (2020). Evaluation of Single and Combined Toxicity of Bisphenol A and its Analogues Using a Highly-Sensitive Micro-biosensor. J. Hazard. Mater. 381, 120908–120916. 10.1016/j.jhazmat.2019.120908 31352154

