# Supplementary material for: Direct and Sensitive Electrochemical Detection of Bisphenol A in Complex Environmental Samples Using a Simple and Convenient Nanochannel-Modified Electrode
Source: Front Chem. 2022 May 26;10:900282. doi: 10.3389/fchem.2022.900282 (PMC9204582; doi:10.3389/fchem.2022.900282)
Supplement: Supplementary file 1 [file DataSheet1.PDF]

TABLES

Table S1. The E_p, I_p and ΔE of electrodes.

	E_{pa}/V	E_{pc}/V	ΔE/mV	I_{pa}/μA	I_{pc}/μA
GCE	−0.1198	−0.1857	65.92	3.084	−3.097
p-GCE	−0.1343	−0.1979	63.58	6.813	−6.022
VMSF/p-GCE	−0.1296	−0.1955	65.92	10.18	−9.449
SM@VMSF/p-GCE	/	/	/	/	/

Table S2. The R_{ct} and R_s of electrodes.

	R_{ct}/Ω	R_s/Ω
GCE	82	110
p-GCE	61	120
VMSF/p-GCE	302	119
SM@VMSF/p-GCE	407	194

FIGURES

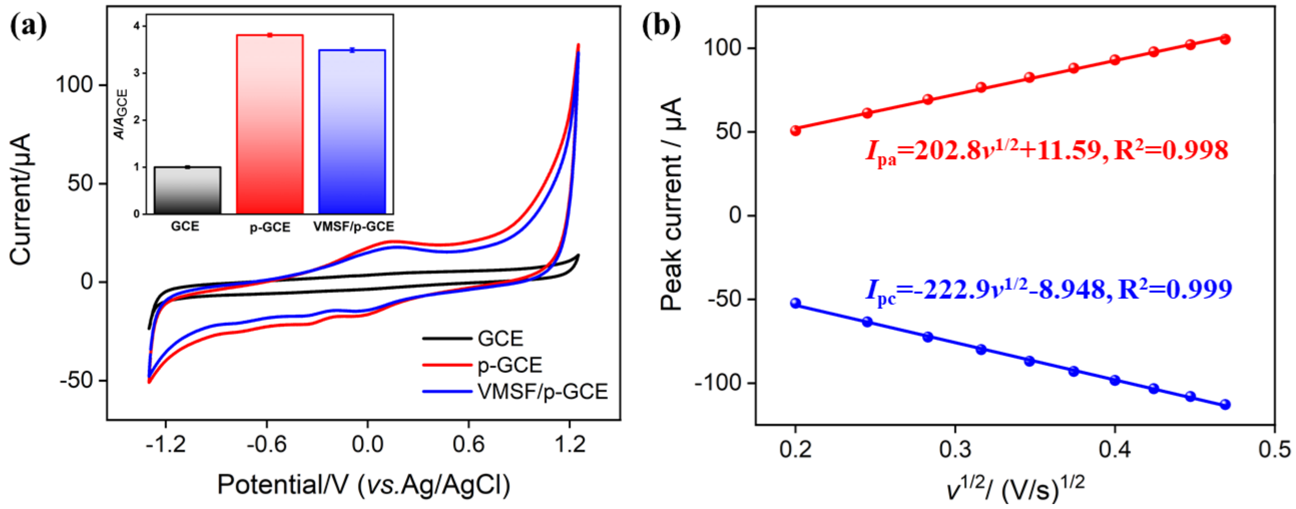

Figure S1. (a) CV curves of GCE (black), p-GCE (red) and VMSF/p-GCE (blue) obtain in PBS (0.1 M, pH=6) saturated with N_2. The inset depicts the normalized electroactive surface area of each electrode. (b) Relationship between peak current *vs*. square root of scan rate derived from the CV curves of GCE obtained in 0.1 M KCl containing 5 mM $K_3[Fe(CN)_6]$.
